# Metabolic model-based analysis of the emergence of bacterial cross-feeding via extensive gene loss

**DOI:** 10.1186/s12918-018-0588-4

**Published:** 2018-06-15

**Authors:** Colin P. McNally, Elhanan Borenstein

**Affiliations:** 1 Department of Genome Sciences, University of Washington, Seattle, WA USA; 2 Department of Computer Science and Engineering, University of Washington, Seattle, WA USA; 3 Santa Fe Institute, Santa Fe, NM USA

## Abstract

**Background:**

Metabolic dependencies between microbial species have a significant impact on the assembly and activity of microbial communities. However, the evolutionary origins of such dependencies and the impact of metabolic and genomic architecture on their emergence are not clear.

**Results:**

To address these questions, we developed a novel framework, coupling a reductive evolution model with a multi-species genome-scale metabolic model to simulate the evolution of two-species microbial communities. Simulating thousands of independent evolutionary trajectories, we surprisingly found that under certain environmental and evolutionary settings metabolic dependencies emerged frequently even though our model does not include explicit selection for cooperation. Evolved dependencies involved cross-feeding of a diverse set of metabolites, reflecting constraints imposed by metabolic network architecture. We additionally found metabolic ‘missed opportunities’, wherein species failed to capitalize on metabolites made available by their partners. Examining the genes deleted in each evolutionary trajectory and the deletion timing further revealed both genome-wide properties and specific metabolic mechanisms associated with species interaction.

**Conclusion:**

Our findings provide insight into the evolution of cooperative interaction among microbial species and a unique view into the way such relationships emerge.

**Electronic supplementary material:**

The online version of this article (10.1186/s12918-018-0588-4) contains supplementary material, which is available to authorized users.

## Background

Most microorganisms in nature do not live in isolation but are rather part of complex communities [1]. The various species that form these communities not only share a common environment, but rather interact with other community members in various ways including competition for extracellular nutrients, cooperation through metabolite cross-feeding, signaling, biofilm formation, and antimicrobial secretion [2, 3]. Such interactions allow community members to impact each other’s behavior and thus play an important role in shaping community structure and activity. A better understanding of how these interactions emerge through ecological and evolutionary dynamics, how they are maintained or lost, and how they impact community-level behavior is therefore crucial for both elucidating the forces that have shaped current natural communities and for designing synthetic communities or targeted modulation of natural communities [4].

Perhaps the most intriguing form of microbial interaction is inter-species cooperation. The prevalence of cooperative interaction is evident from the large number of microbes that cannot be individually cultured, suggesting that they are reliant on symbiotic interactions with other members of their communities [5]. In the context of metabolism, cooperation often takes the form of cross-feeding, where one species secretes metabolites that other species uptake and utilize. Indeed, metabolic cross-feeding has been found to occur in a wide variety of environments and between diverse species [6], often benefiting both partners [7]. For example, *Bifidobacterium* species in the gut microbiota regularly cross-feed fermentation products and partial digestion byproducts of polysaccharides to butyrate-forming bacteria [8]. Furthermore, evidence suggests that metabolic cooperation drives species co-occurrence in diverse microbial communities [9].

Importantly, however, metabolic cooperation is not limited to complex communities and has also been demonstrated in small two- or three-species communities, such as those occupying various insect hosts. For example, it was shown that the two endosymbionts that occupy a sharpshooter insect (and the insect host) each lack necessary steps of several amino acid synthesis pathways, and consequently only when the three organisms grow together can they synthesize the entire complement of amino-acids [10, 11]. Similarly, it was shown that two endosymbiotic bacteria that inhabit a *Cicadoidea* host had recently diverged into 3 species, with metabolic complementarity between the two recently split lineages [12]. In such small communities, cooperation likely emerges not through selection (e.g., via the Black Queen hypothesis [13], where loss of metabolic capabilities and development of dependence has a selective advantage [14–16]), but rather by chance as a consequence of extreme genome reduction [17]. Indeed, most insect endosymbionts have extremely small genomes (some of which are the smallest bacterial genomes known) that are majorly reduced compared to their closest free-living relatives [18]. Moreover, such tightly coupled minimal metabolic systems, where two or three species strongly depend on each other for survival, can be viewed as an idealized model of microbial cooperation and provide insight into the evolution of cooperative interactions [19].

Notably, however, despite the prevalence and diversity of cooperative endosymbiont systems, the process through which extensive long term genome reduction leads to metabolic cross-feeding and the mechanisms involved in such evolution are not clear and are challenging to study. Experimental evolution studies, for example, have demonstrated the emergence of cooperative interactions between divergent polymorphic sub populations [20, 21], but are generally limited in both the duration of evolution and the number of replicates. These limitations hinder a systematic and comprehensive study of long term genomic reduction or identification of general principles in the emergence of species interaction. On the other hand, theoretical and computational models have been broadly useful for studying microbial communities [22, 23], and indeed several recent studies have used computational models to specifically address the evolution of cooperation [24, 25]. Such studies have allowed long time scales to be easily modeled and have produced useful insights into genetic and environmental determinates of cooperation, but tend to explicitly model interaction in a non-mechanistic manner. Such models may therefore fail to capture the mechanisms underlying metabolic cross-feeding and the processes through which genome reduction can give rise to such mechanisms.

To address this gap, in this study, we utilized a model of microbial evolution over a *long time scale* coupled with a *mechanistic model* of multi-species microbial metabolism and growth. Our model is inspired by a previous study that modeled reductive evolution of a single endosymbiont species and investigated how historical contingency and timing of gene deletions affects future genome reduction [26, 27]. In the work we present here, we extended this evolutionary framework to a co-culture model of two species, using a mechanistic model of microbial growth in co-culture based on a multi-species genome-scale metabolic modeling approach [28]. Such a framework allows us to simulate long evolutionary trajectories, to investigate metabolic mechanisms on a genome-level, and to generate a large number of simulated trajectories for inferring general principles in the evolution of metabolic interaction.

We specifically aim to examine whether species interaction can emerge in a simple multi-species community without explicit selection for it, which mechanisms can drive a selfishly evolving species to support a dependent species, and how the architecture of the metabolic and genetic networks affects the evolution of such interactions. Notably, we do not model the process by which an evolving population bifurcates into multiple subpopulations, but rather explicitly assume the community harbors two evolutionary isolated species (e.g., following an initial split), each undergoing an extreme reductive evolution process (such as the one experienced by insect endosymbionts). This assumption allows us to examine evolutionary trajectories and species interactions between two well-defined linages in a fixed community context (and see also Discussion). We further assume that these two species co-exist over a long time scale, without one out competing the other. It is also important to note that we do not necessarily aim to model the evolution of any specific species or community, nor the evolution of any specific metabolic pathway, but rather to examine general principles and patterns that may be observed when the evolution of species’ metabolic networks are driven by such a reductive evolutionary scheme. Using this framework, we simulated thousands of independent evolutionary trajectories, tracked the emergence of metabolic cross-feeding, and carefully analyzed the evolving species. Our findings shed light on the evolution of species interactions and could inform future effort to construct stable microbial communities for medical, agricultural, and industrial applications.

## Results

### A framework for modeling the evolution of species interactions

**Fig. 1:**
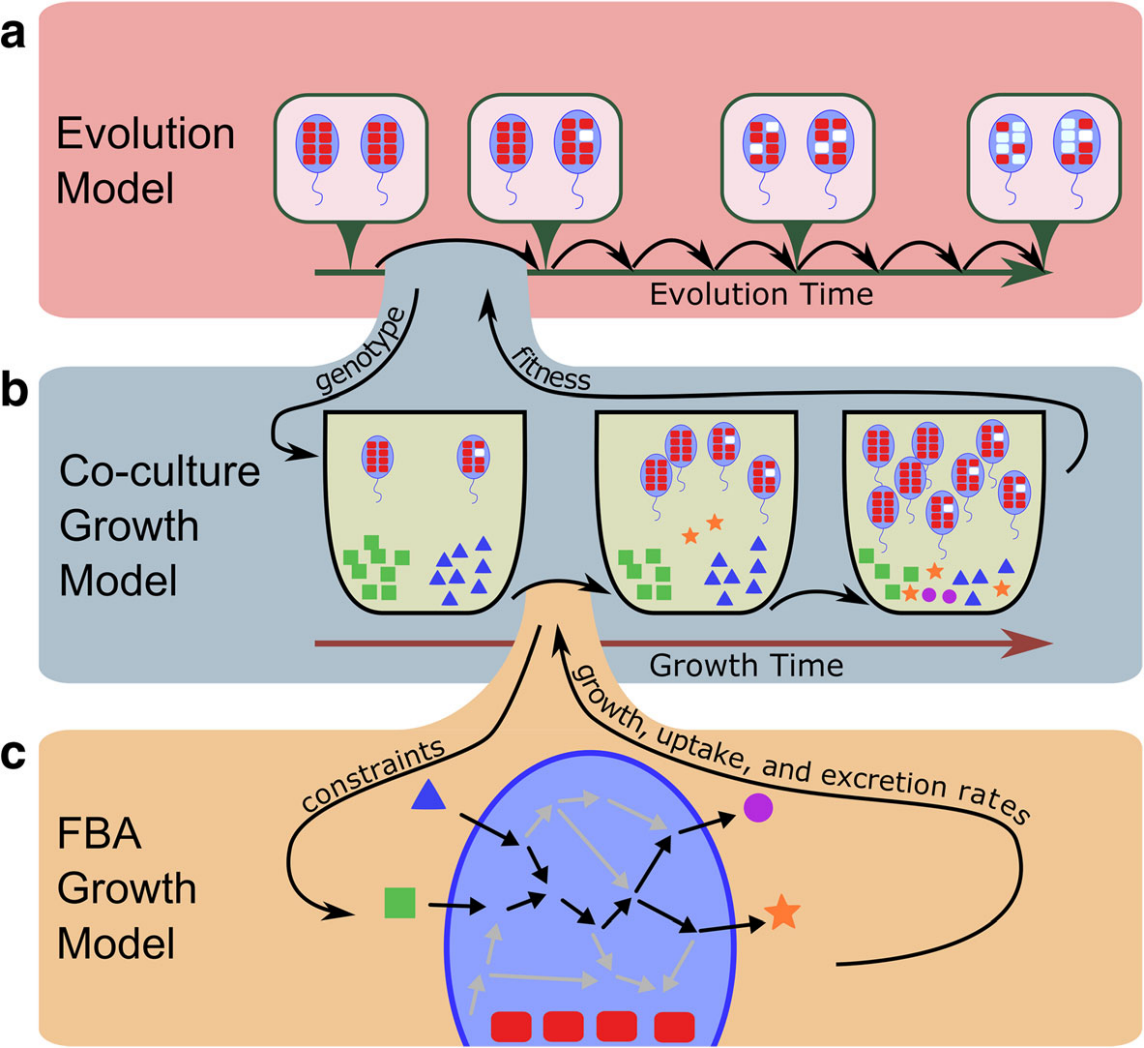
A framework for modeling the evolution of species interaction. **a** To model reductive evolution, genes are iteratively chosen at random as candidates for deletion, the fitness effect of their deletion is evaluated (using a co-culture growth model; panel (**b**)), and if the fitness effect is relatively small, these genes are deleted. **b** The co-culture growth model simulates the growth of the two species in a shared environment, and is based on a previously introduced dynamic multi-species model [28]. This model iteratively infers the behavior of each species in the shared environment based on an FBA approach (panel (**c**)). The predicted growth of each species and the predicted rates at which it uptakes and excretes various metabolites are used to update the abundances of species in the co-culture and the concentration of metabolites in the shared environment over time. **c** An FBA model is used to predict the growth of each species in a given environment based on the set of metabolic reactions and constraints encoded by the species and the concentration of metabolites in its environment

To study the emergence of metabolic species interaction in bacteria we developed a computational framework that integrates models of microbial co-evolution, metabolic activity, and ecological interaction (Fig. 1). Briefly, in this framework, we model a community comprised of two generalist species growing in a shared environment (and that can therefore exchange metabolites) that go through a reductive evolution process. In our model, evolution is an iterative process (as in [26]) in which a gene is first chosen at random from either of the two species for deletion (Fig. 1a). The fitness effect (measured as the change in growth rate) of losing that gene in the context of the community is calculated using a co-culture metabolic model (described below). If the decrease in fitness to the species losing this gene does not exceed a predefined threshold the deletion is assumed to fix; otherwise the deletion is assumed to be selected against and is reverted. Importantly, during the course of this co-evolutionary process, the presence of each of the two species in the community can markedly impact the evolution of the other (and specifically, the set of genes that can be deleted). This process repeats until no more genes can be deleted from either species.

To model growth in co-culture and to determine the fitness consequence of gene deletions while accounting for the way the presence of one species in the community may impact the fitness of the other, we used a co-culture metabolic modeling framework [28]. This framework employs dynamic Flux Balance Analysis (FBA) [29] to predict the metabolic activity and growth of two species in a shared environment over discrete time points (Fig. 1b). At each time point and for each species in the co-culture, this framework uses a genome-scale metabolic model of the species (based on its metabolic capacity as determined by the set of genes present in its genome), the current concentration of metabolites in the environment, and a flux balance analysis to predict the species’ behavior, including its growth rate and the rate at which it imports and excretes various metabolites [30] (Fig. 1c). The estimated growth rates of the two species are then used to update the abundances of the species in the community and the predicted uptake and excretion fluxes are used to update the concentration of metabolites in the shared environment. Growth is simulated over several time points and the growth rates of each species at the last time point are used as proxies for their fitness. This co-culture model accounts for gene loss only through the resulting loss of metabolic capacities, and therefore includes only metabolic genes and ignores the potential consequences of loss of other genes.

**Fig. 2:**
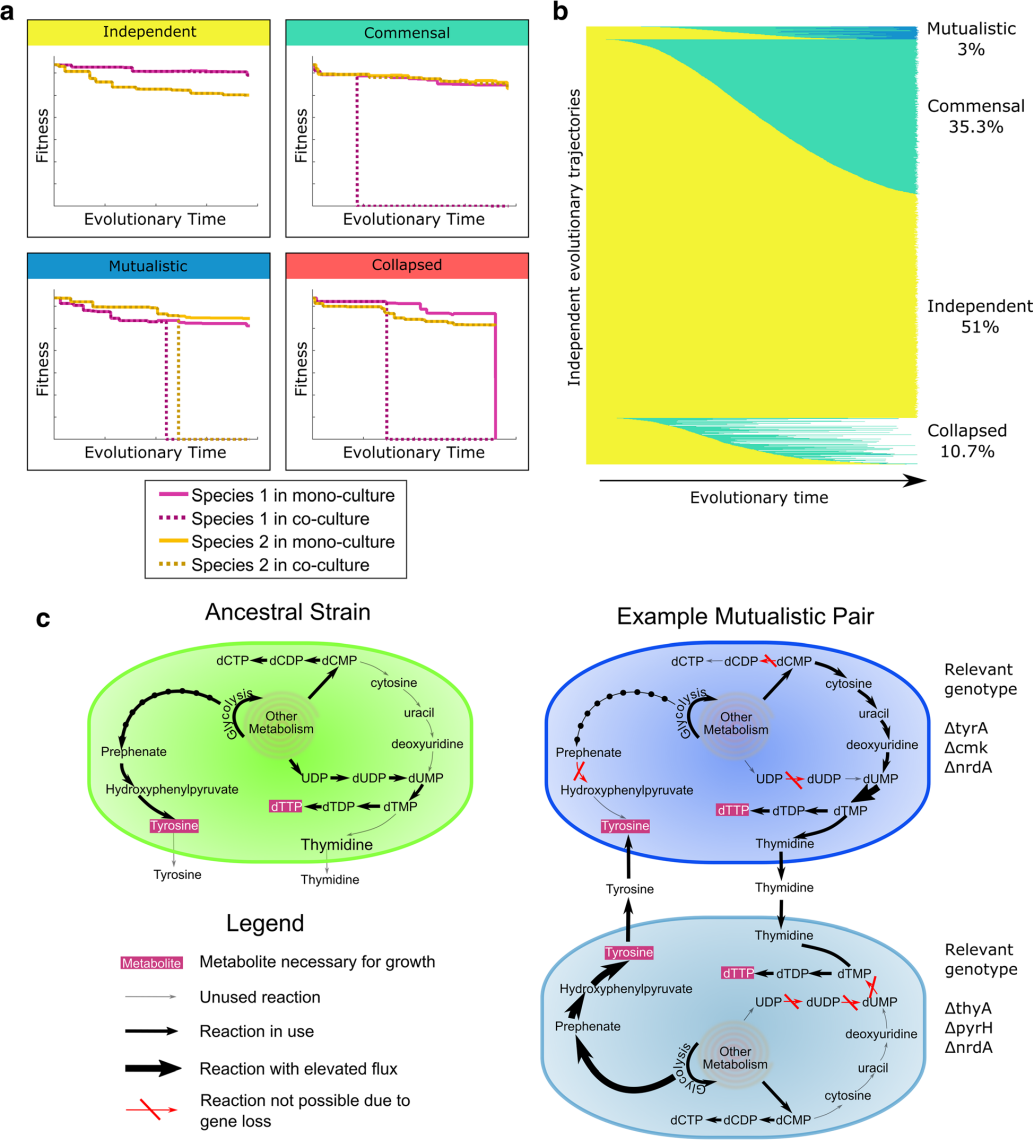
Metabolic interactions and their emergence over time. **a** Evolutionary simulations could result in one of four unique outcomes, determined by the ability of evolved species to grow in mono-culture and co-culture. Plotted are examples of each of these four outcomes, illustrating the fitness of each of the two species in mono-culture and in co-culture over evolutionary time. **b** The changes in interaction type over time for all 16,317 simulation runs. Each horizontal bar represents a single simulation run, and the color corresponds to the interaction type using the same colors as the titles in panel (**a**). **c** Example of an evolved mutualistic community**.** In the ancestral species tyrosine is produced through the shikimate pathway and dTTP is produced from UDP. In this example evolved mutualistic community, deletions in both species have led to obligate cross-feeding of tyrosine and thymidine. The relevant gene deletions and their impact on metabolic fluxes in each species are highlighted

To classify the interaction between the two species in each community and at each evolutionary step, we also simulated and evaluated the growth of each of the two species in isolation (i.e., in mono-culture). We define a species as being dependent on its partner if it can grow in co-culture but not in mono-culture. We further distinguish between three possible types of interaction a given community can exhibit: (i) *Independent* – neither species is dependent on the other, (ii) *commensal* – one species (*‘dependent’*) is dependent on the other species but the other (*‘provider’*) is not, and (iii) *mutualistic* – both species are dependent on each other. Note that we use the terms ‘commensal’ and ‘mutualistic’ to describe the presence/absence of dependency and whether dependency is unidirectional or mutual, ignoring the more subtle distinction (and pertaining ecological definitions) of whether species harm each other or not. The observed interaction type at the end of the simulation run (i.e., when both species reach minimal genomes) was used to label each evolutionary trajectory (Fig. 2a). Notably, in some cases, one of the two species can go through a catastrophic drop of fitness (> 50%) even in co-culture (e.g., due to a change in the *other* species’ behavior that limits the availability of a metabolite it requires). In such cases, that species was considered to have gone extinct and the simulation was labeled as a collapsed community. A detailed description of the framework is provided in Methods.

### The emergence and prevalence of metabolic species interaction

We used the framework described above to simulate 16,317 independent evolutionary trajectories of a simple community comprising two generalist species that go through a reductive evolution process (see Methods). As noted above, we assumed that the community composition is fixed as a two genotype community, with no new species migrating into the community and no standing genetic diversity. In each simulation, we initialized the community with two identical *E. coli* strains (as a generalist model species [26]), representing the evolution of two obligate symbionts that may have diverged from a common ancestor [12].

Surprisingly, although our framework does not impose an explicit pressure toward species interaction, we found that a substantial fraction of simulations resulted in a community with some sort of metabolic dependency between the two species. Specifically, 35.3% of the simulations ended with a commensal community, and 3% of the simulations ended with a mutualistic community (Fig. 2b). In 10.7% of the simulation, the community collapsed as described above. The remaining 51% of simulations ended with independent communities.

We additionally examined the impact of various simulation settings on the prevalence of different interactions types. We first tested the effect of using different fitness cutoff values. In natural communities the strength of selection against deleterious gene deletion reflects multiple factors, ranging from population size to environment stability, which therefore indirectly affects the likelihood of emergent cooperation. Indeed, we found that using different fitness cutoffs for allowing deleterious gene deletions to fix affected the ratio of the different interaction types, with a more stringent cutoff resulting in more independent communities and a less stringent cutoff resulting in more commensal and mutualistic communities (see Additional file 1: Supporting Text). We next examined the impact of evolving communities in rich rather than minimal media. A richer media may hinder the emergence of species interactions since useful metabolites could be obtained from the environment rather than via cross-feeding. Exploring the impact of several types of richer media, including media with additional carbon sources or with various sets of amino acids, we found that indeed such media generally increased the prevalence of independent communities, though the specific effect was different for different media types, with varying balance between commensal, mutualistic, and collapsed communities (see Additional file 1: Supporting Text). Finally, we examined the impact of different gene deletion strategies, including using different gene loss rates by the two species or allowing deletion of more than one gene at a time. We found that these strategies generally had little impact on the prevalence of various interaction types, although deleting multiple genes at each iteration did increase the prevalence of collapsed communities (see Additional file 1: Supporting Text).

### An example of an emergent cross-feeding interaction

Before exploring large-scale patterns concerning emerged mechanisms involved in species interaction, we set out to characterize in detail one evolved mutualistic community as an example of the kind of metabolic interaction that could emerge and the gene deletions that underlie such an interaction. In this community, the two species (arbitrarily referred to below as species A and species B) had retained only 306 and 304 genes respectively, compared to 1260 genes in the ancestor species. Per our definition above, these two minimal species could still grow in co-culture (albeit at only 78 and 73% of the ancestor’s growth rate, respectively), but neither could grow in mono-culture. Analyzing the evolved metabolic dependency of these species (Methods), we found that species A became dependent on tyrosine (and could grow on the initial medium once tyrosine was added) and that species B became dependent on thymidine (and similarly could grow on the initial medium once thymidine was added).

We further examined the fluxes through the metabolic models of the evolved species and compared them to the fluxes observed in the ancestor species, to identify the specific gene deletions that gave rise to these dependencies (Fig. 2c). We found that species A became dependent on external tyrosine due to a loss of the gene *tyrA*, which is necessary for tyrosine synthesis [31]. Indeed, species A’s loss of *tyrA* occurred at the exact same point in the evolutionary trajectory as its loss of the ability to grow in mono-culture. Similarly, Species B became dependent on external thymidine due to a loss of the gene *thyA*, which is necessary for dTMP synthesis [32]. We were also able to identify the evolved mechanisms that allowed each of the two species to excrete the metabolite necessary for growth of the other species. Specifically, species A started excreting thymidine due to a loss of the gene *cmk*, which is necessary to phosphorylate CMP to CDP [33]. The loss of several other reactions prevented species A from converting CMP to cytidine, uridine, uridine monophosphate, excreted uracil, or thymine, which resulted in species A only being able to eliminate excess CMP by converting it to thymidine and excreting it. Notably, a *cmk* deletion in *E. coli* has been shown experimentally to result in 30-fold elevated CMP and dCMP pools relative to wild-type [33]. Species B similarly excreted tyrosine due to an overproduction of this metabolite following a complex combination of gene losses that resulted in elevated activation of the pentose phosphate pathway and converting excess erythrose-4-phosphate into tyrosine. This example highlights the complex mechanisms that may be involved in the evolution of metabolic species interaction and how the architecture of the metabolic network could facilitate such interactions.

### Metabolite cross-feeding and dependency in evolved pairs

**Fig. 3:**
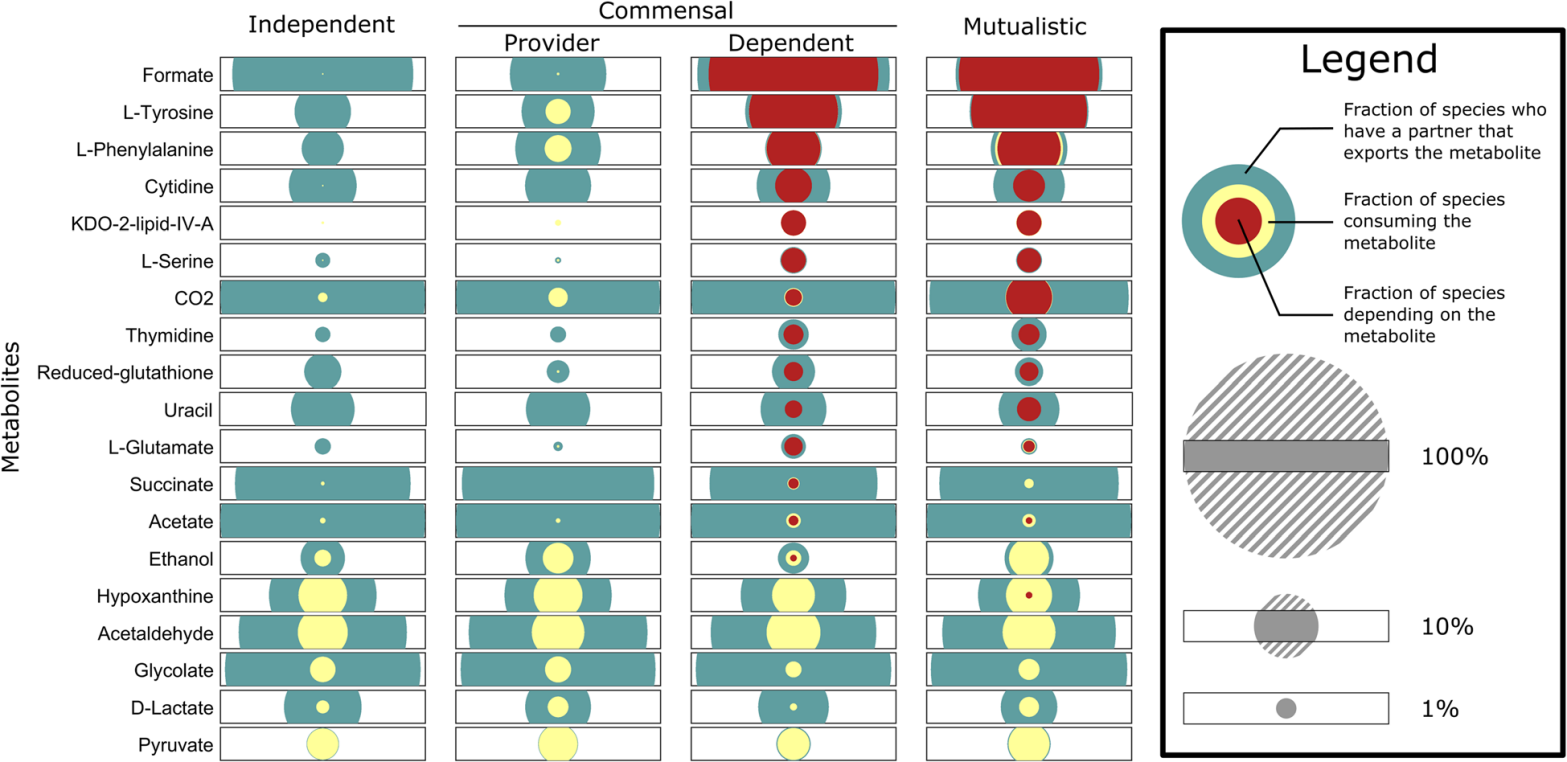
Frequencies of metabolites’ availability, cross-feeding, and dependence. The frequencies of metabolites’ availability, cross-feeding, and dependence are shown for species of each interaction type and for each metabolite. Each set of nested circles shows the frequency at which the given metabolite is produced by their partner species and hence available for uptake (blue), the frequency at which this metabolite is utilized by the species through cross-feeding (yellow), and the frequency at which this metabolites is depended on (red). The area of the circle scales with the frequency, but for visualization purposes the portions of the circle extending beyond the rectangular box are not shown. Only metabolites that are depended upon at least 10 times or consumed at least 30 times are shown

After characterizing one cooperating pair in detail, we set out to examine the complete set of communities evolved by our model, focusing initially on identifying the metabolites underlying emergent species interactions (Methods). We found that the majority of dependent species (94.3%) required only a single essential metabolite to be cross fed from their partner, with only a small fraction of dependent species requiring two or three such metabolites (5.6 and 0.2% respectively), and no species requiring more than three. Formate was the most common essential metabolite (68.7% in commensal dependent species; Fig. 3), followed by Tyrosine (18.8%) and Phenylalanine (6.9%). Notably, the dependence on a single (or very few) metabolites reported above contrasts observations made in several insect symbionts systems where cooperating symbionts exchange multiple essential compounds (and see Discussion below), yet the exchange of aromatic amino-acids is in agreement with cross-fed metabolites often observed in such systems [10, 34].

Complete dependence on cross-fed metabolites (such as those identified above) is the most defining feature of species interactions in these communities, but may represent an extreme form of interaction. Clearly, cross-feeding can be beneficial to a species even when it is not essential for growth, and in fact this form of cross-feeding may be a common precursor state of complete dependence. Examining secretion and uptake fluxes (Methods), we indeed identified multiple metabolites that are being cross-fed but are non-essential to growth (Fig. 3, yellow circles). Many of these non-essential cross-fed metabolites were rarely if ever depended on (such as acetaldehyde and pyruvate) and were observed at similar frequencies across all interaction types. Interestingly, however, we also detected non-essential cross-feeding of metabolites that were commonly depended upon (such as tyrosine and phenylalanine; Fig. 3), but these were rare in independent communities and occurred surprisingly often in commensal communities where *providers* were cross-fed such metabolites by the dependent partner. This finding suggests that species cooperation may involve two species that evolve a similar metabolic strategy (and therefore have the potential to both excrete and utilize a similar set of metabolites). In such cases, cross-feeding is likely to emerge, first as a non-essential process, which may later evolve into species commensal or mutual dependence. To confirm this hypothesis, we specifically examined, for each dependent species, the time that elapsed from when this species started consuming a metabolite via cross-feeding to when it became dependent on that metabolite. In most cases dependence does not immediately follow cross-feeding, and there is often a substantial delay between cross-feeding and dependency (Additional file 1: Figure S1).

Clearly, uptaking a metabolite is only possible if the partner species is producing that metabolite and excreting it to the shared environment, thereby providing an opportunity for cross-feeding. We additionally quantified the frequency and time at which such opportunities arose, regardless of whether the metabolite was consumed or not (Fig. 3, blue circles). We found that metabolites vary greatly in the frequency at which they are excreted, and in a way that is not fully correlated with the frequency at which they are cross-fed or dependent on. For example, various metabolites, including cytidine, succinate, and acetate, are excreted at relatively similar frequencies in all interaction types, suggesting that dependency on these metabolites is not limited by their availability. Conversely, other metabolites such as serine and thymidine are rarely excreted in independent communities, suggesting that the availability of these metabolites often leads to cross-feeding and dependency on them. Most importantly, while cross feeding often started almost immediately after the metabolite was available (in cases in which it occurred; see Additional file 1: Figure S1), in many cases evolving species failed to utilize available metabolites, thus completely missing cross-feeding (both essential and non-essential) opportunities (Fig. 3). This finding implies an intriguing dichotomy where available opportunities are either utilized immediately or are not utilized at all, potentially due to evolutionary constraints.

### The genomic basis of evolved species interactions

**Fig. 4:**
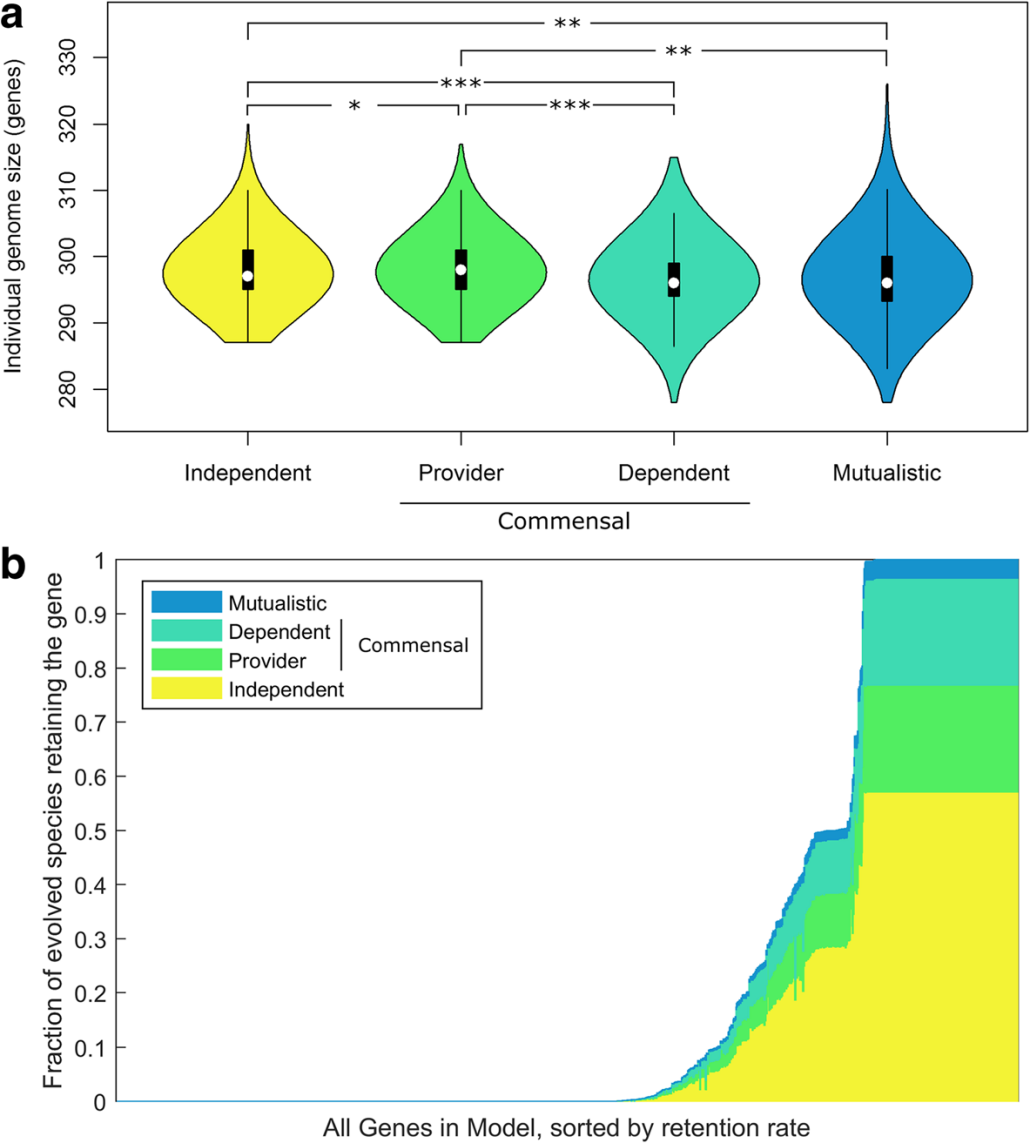
Genome size and gene retention frequency in evolved genomes. **a** Distributions of the genome size of evolved species from each interaction type. (*: *P* < 10^− 3^**;** **: *P* < 10^− 9^**;** ***: *P* < 10^− 30^). **b** The distribution of retention rates of different genes in the model. Each gene is plotted as a vertical bar with height equal to the fraction of species in non-collapsed simulations that retained it, and the genes are sorted in ascending order of this overall retention rate. Each bar is colored by the fraction of species retaining that gene that are in each of the different interaction types

Our mechanistic model of microbial metabolism allows us to move beyond a phenotype-level description of evolved communities and to directly investigate patterns of genome evolution and identify genomic mechanisms involved in species interactions. We first examined the number of genes that were retained or lost in different simulations to explore the relationship between genome size (in terms of the number of genes retained) and species interaction. Surprisingly, with the exception of collapsed communities, evolutionary trajectories exhibited a markedly low variation in the total number of genes retained, with an average of 297.6 ± 4.4 genes retained in each species. Yet, we found that both dependent and mutualistic species had slightly but significantly smaller genomes compared to independent species (*P* < 10^− 30^ and *P* < 10^− 9^ respectively; two sample t-test; Fig. 4a), while provider species had slightly but significantly larger genomes than independent species (*P* < 0.001). Similarly, within commensal communities, the genomes of dependent species were slightly but significantly smaller than the genomes of provider species (*P* < 10^− 30^). Moreover, while these differences in *average* genome size were generally very small (often less than a single gene; see Discussion), the differences between the *smallest* genomes observed in the dependent or mutualistic species and the smallest genomes observed in provider or independent species was much larger (Fig. 4a). These results are consistent with the idea that cross-feeding allows dependent species to lose genes they would not be able to lose otherwise [11, 35]. Interestingly, provider species had on average slightly larger genomes than independent species (*P* < 10^− 3^). This could suggest that provider species retain additional genes (i.e., that are generally lost in independent species), likely as a result of constraints associated with earlier gene deletions. Such genes and the extended metabolic capacities with which they endow provider species may in turn give rise to metabolite overproduction. Alternatively, additional genes may be retained by provider species to allow overproduced metabolites to be excreted outside the cell. Additional simulations further demonstrated that the genome size of independent species was similar to the genome size of species evolving in mono-culture conditions (see Additional file 1: Supporting Text).

**Fig. 5:**
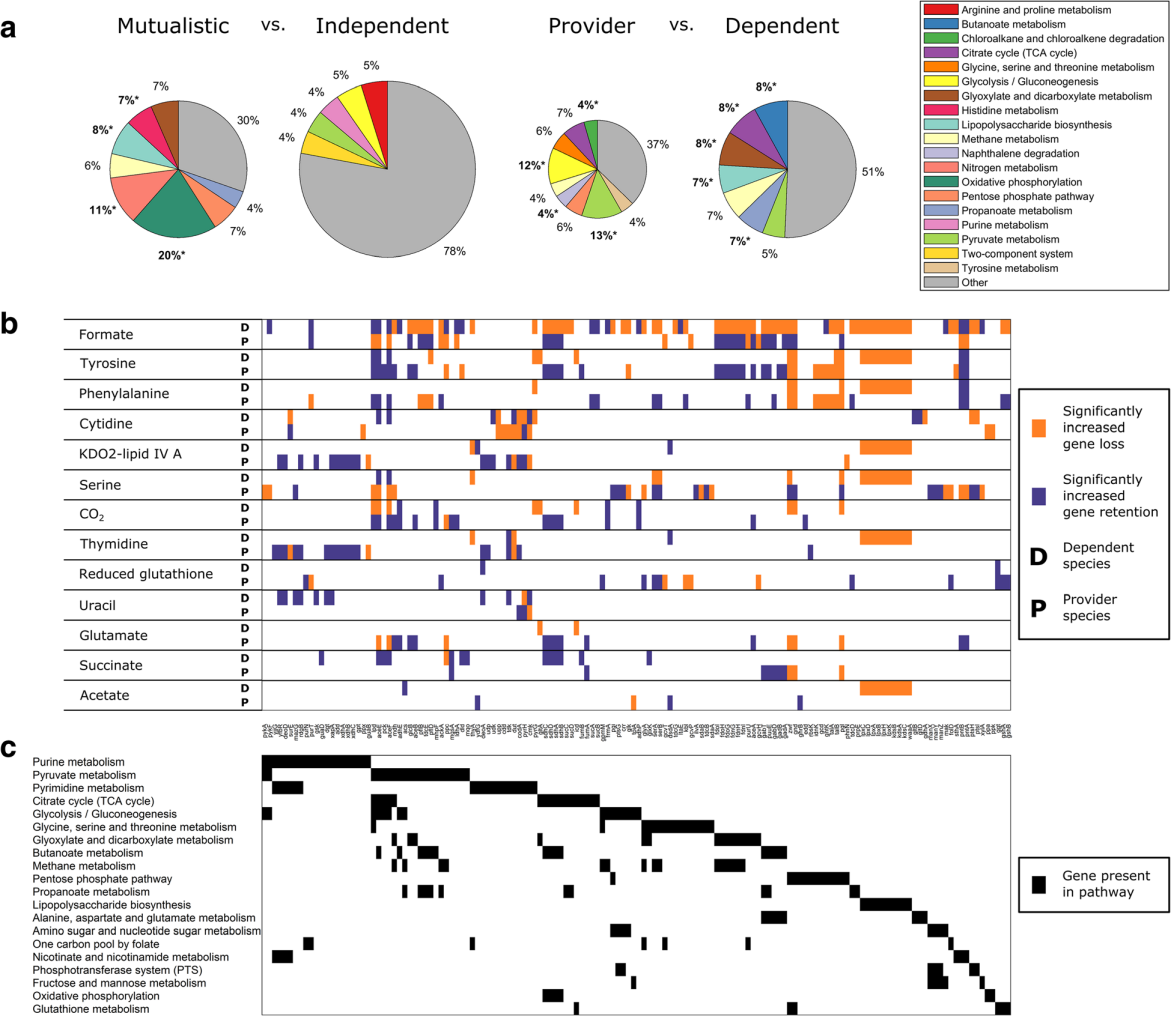
Genes and pathways associated with species interaction and cross-feeding phenotypes. **a** The fraction of genes whose deletion is associated with each interaction type that are assigned to each pathway. The overall size of the pie indicates the number of genes associated with that interaction type. Pathways that are significantly enriched among this set of genes are highlight (bold font and asterisk). Only pathways that contain > 4% of the genes are listed (the rest are pooled into the ‘Other’ category) **b** Associations between providing or depending on specific metabolites and deletion or retention of specific genes. Orange (purple) bars indicate genes that are lost (retained) significantly more often in species that provide or depend on a specific metabolite compared to independent species. Genes are sorted by membership to pathways (see (**c**)), and only genes present in at least one pathway are shown. **c** Pathway membership of genes. Black bars indicate that a gene belongs to that pathway. Pathways are sorted by the number of genes assigned to them, and only pathways with five or more assigned genes are shown

Notably, examining the genes retained across the evolved species, we found that of the initial 1260 genes present in the ancestral species, 560 were always lost and 149 were always retained, with only 551 genes being retained at intermediate frequencies (Fig. 4b, Additional file 2: Table S1A). The specific subset of these 551 genes that were retained in each evolved species therefore determines the types of interaction that emerged, and indeed a statistical analysis was able to detect specific genes whose retention or loss was associated with specific types of interaction (Fig. 5a, Additional file 1: Supporting Text, Additional file 2: Table S1B). Further examining how similar, on average, are the sets of genes retained between the two partners in each community, we also found that cooperating partners (i.e., from mutualistic or commensal communities) were less similar to each other than independent partners, suggesting that the evolution of metabolic dependency is associated with a process of functional diversification (Additional file 1: Supporting Text).

To better understand the diversification between partners in commensal communities (in which the two species can be labeled clearly as dependent and provider and therefore the direction of dependency is clear), we compared the set of genes retained in providers vs. those retained in dependents, identifying 80 genes that are more frequently retained in the provider and 41 that are more frequently retained in the dependent species. For example, *pflA*, a pyruvate formate lyase, was retained in 55.5% of providers but only 16.8% of dependents, whereas *aceE* and *aceF*, both components of the pyruvate dehydrogenase complex, were retained in 81.1% in dependent species and only 19.0% in provider species (these genes were also those with the greatest differential retention rate). We additionally identified a set of 263 gene *pairs* that are significantly exclusively-retained in commensal communities (i.e., the dependent species retained the first gene when the provider lost the second gene or vice-versa more often than expected by chance, Additional file 2: Table S1C; Methods). Interestingly, this set was enriched for gene pairs that shared a pathway annotation (*P* < 10^− 4^; permutation-based test), suggesting complementation at the pathway level. To finally examine the *dynamics* of gene deletion events in these commensal communities and to reveal key evolutionary steps on the route to cross-feeding, we further used a permutation-based analysis to identify instances where a gene in one species tended to be deleted only after another gene was deleted in the partner species (Methods). Our analysis discovered several such ordered events, where a deletion leading to dependency on a given metabolite in one species could occur only after deletions that promoted over-production and excretion of that metabolites occurred in the second species (Additional file 1: Supporting Text and Fig. S2).

### Linking genome evolution to metabolite cross-feeding

Having identified both the metabolites involved in cross-feeding and the genes involved in the emergence of species interaction, we finally turned to examine the association between specific gene retention or loss events and the cross-feeding of specific metabolites. Towards this end we again considered the set of all commensal communities and, for each of the 13 cross-fed metabolites that were depended upon in at least 10 communities, identified genes whose retention or deletion are significantly correlated with the excretion of this metabolite in the provider or with the dependency on this metabolite in the dependent (Methods). In total we identified 310 gene-metabolite associations (including 204 retentions and 106 deletions) in provider species associated with essential metabolite excretion, and 315 gene-metabolite associations (114 retentions and 201 deletions) in dependent species associated with metabolite dependency (Fig. 5b, Additional file 2: Table S1D; χ^2^ test, 1% FDR). In total, retention or loss of 194 of the 551 variable genes was significantly associated with excretion of or dependence on at least one metabolite, with many genes associated with the excretion of or dependence on multiple metabolites. This finding demonstrates how the interconnectedness of the metabolic network impacts the evolution of species cooperation, wherein the loss of a key gene could give rise to multiple metabolic phenotypes.

Interestingly, certain genes were associated with both the excretion of a metabolite by the provider *and* with the dependence on that same metabolite in the dependent species (Fig. 5b). For example, retention of *tyrP* and *aroP* (aromatic amino acid transporters) were associated with both providing tyrosine and dependence on tyrosine, as cross-feeding of that metabolite required that both species in the pair could exchange it with the shared environment. In other cases the same gene was associated with a metabolite being excreted by the provider and utilized by the dependent but in a different direction. For example *serA* and *serB* – genes involved in serine biosynthesis – tended to be lost in species dependent on serine, but retained in species producing it. In rare cases there were genes whose loss was associated with both dependence and providing of a specific metabolite. For example, the loss of the gene *codA* was associated with both excretion of cytidine by providers and dependency on cytidine in dependent species (and see our analysis of that gene above). Combined, these associations suggest that metabolite cross-feeding can evolve via multiple mechanisms and that such mechanisms are often metabolite- and gene-specific.

Finally, to further explore metabolic mechanisms that may be involved in cross-feeding, we examined the pathways to which genes associated with various metabolic phenotypes are assigned (Fig. 5c). Many pathways reflect expected associations, such as the pyrimidine metabolism pathway (that includes many genes whose loss or retention is associated with cytidine cross-feeding) and the pyruvate metabolism pathway (that includes many genes associated with formate cross-feeding). In other cases the link between deletion of genes from a specific pathway and metabolic cross feeding is less obvious. For example, cross-feeding of thymidine and uracil, both pyrimidines, is associated with the deletion/retention of several genes in the pyrimidine metabolism pathway but also with a number of genes in the purine metabolism pathway. This could suggest that cross feeding of these metabolites is driven by a metabolic overflow that originates in the purine metabolism pathway but results in excess nucleotides being converted to pyrimidines before being excreted as waste. More generally, the association of genes from multiple pathways with cross-feeding of a single metabolite further highlights how the interconnectedness of the metabolic network gives rise to non-trivial links between gene deletion and cross feeding.

## Discussion

In this study we investigated the potential for metabolic interactions to emerge between two species inhabiting a shared, constant, nutritionally limited, and isolated environment and undergoing extensive gene loss. We found that cross-feeding interactions emerged frequently in these settings, and that such evolved communities exhibited diverse, multifaceted, and non-trivial metabolic interactions that were not necessarily optimized at the community level; useful metabolites were often excreted by one species but not utilized by its partner and other metabolites were cross-fed without evolving complete dependency. Such “suboptimal” interactions and missed metabolic opportunities are a reasonable outcome of selfish species evolution in the absence of explicit selection for interaction, and could also occur in natural communities. Another potential contributor to such suboptimal interactions is the interconnectedness of different metabolic phenotypes. This interconnectedness may also account, for example, for the relatively frequent occurrence of provider species utilizing metabolites excreted by their dependent partners, where the gene retention and loss events that cause a dependent relationship in one direction may also facilitate emergence of a reciprocal cross-feeding relationship.

Indeed, our analysis has demonstrated that genes were often associated with the excretion and/or production of multiple different metabolites and that the excretion and/or production of each cross-fed metabolite generally involved combinations of multiple deletions. This ‘many-to-many’ mapping between gene loss events and emerged interactions is perhaps not surprising given the coupling between different pathways induced by the architecture of the metabolic network. With each gene deletion metabolic fluxes are redistributed across the network, ultimately rendering the link between gene deletions and cross-feeding of specific metabolites non-trivial and challenging to understand. This observation can also be viewed as an instance of genetic epistasis, where the deletion of two (or more) genes results in an unexpected and non-additive behavior that cannot be easily explained by the cumulative effect of the deletion of each gene in isolation. Moreover, in this context, our framework highlights an exciting extension of genetic epistasis, wherein unexpected and non-trivial interactions between gene deletions can be observed when the two genes are encoded by two different community members (and see [36]). Future work can further delineate and explore this form of multi-species, community-level epistasis.

Interestingly, in our simulations, metabolic *dependency* usually involved a single metabolite, while real world mutualistic endosymbionts often exchange and are dependent on multiple metabolites [10]. One potential explanation is that in our model bacterial growth is optimized (given the metabolic capacities encoded by their reduced genome), whereas in reality extreme genome reduction likely impacts cell regulation and control of growth. Put differently, while generally a reduction in genome size is likely associated with a reduced set of metabolites the cell can synthesize, such disrupted regulation (e.g., via extreme loss of non-metabolic genes) may potentially cause cells to excrete a larger variety of the metabolites they still synthetize and these could be beneficial to their partners. This growth optimization may also account for the relatively small difference in genome size observed in our simulation between dependent and independent species. Our analysis also suggests that the likelihood of missed metabolic opportunities may vary across metabolites, with some metabolites (e.g., cytidine, succinate, and acetate) being excreted at relatively similar frequencies in all interaction types and others (e.g., serine and thymidine) being rarely excreted in independent communities.

Our findings additionally demonstrated how functional diversification leads to metabolic cooperation, where each species retains certain metabolic capacities that the other species has lost. Given a diversification process, it is interesting to speculate about what causes one community to evolve a commensal interaction and another to evolve a mutualistic interaction. We found, for example, that provider species had on average a slightly larger genome than independent species, suggesting that a provider state is the outcome of more constrained evolutionary trajectories that end with larger minimal genomes (e.g., due to early gene deletion events that render other genes essential for growth). This could be the result of providers being forced to eliminate flux overflows through longer pathways that results in more useful waste metabolites being excreted. Moreover, examining the total number of metabolites being excreted by each species, we found that dependent species in fact tend to excrete more metabolites at the beginning of the evolutionary process (see Additional file 1: Supporting Text and Figure S3), potentially suggesting that early ‘wasteful’ metabolic strategies may contribute to the evolution of dependence. Another interesting outcome of our results was the dichotomy observed when a new metabolite became available, with species either starting to consume it immediately and later becoming dependent on it or never consuming it at all. These missed opportunities seem to be examples where the evolutionary events that occurred before the availability of the metabolite precluded utilization of that metabolite by potentially losing the necessary transporter or other reactions necessary for uptake. With this in mind, the non-essential cross feeding observed in commensal communities may simply represent communities that were on the path toward mutualism, but where cross-feeding emerged too late in the evolutionary process when the providers have already lost genes that would be necessary for dependence. This suggests a role for historical contingency in the emergence of cross-feeding, though its importance compared to chance and the extent to which it limits accessible states in co-evolution settings will need to be further examined [26, 37].

Despite these exciting results, there are clearly some caveats in the framework used in this work. For example, our framework assumes that bacteria grow selfishly, and accordingly cross-feeding often requires extensive gene deletions to force excretion of cross-fed metabolites. In reality bacteria can be leaky and release metabolites into their environment even without mutations [4, 13]. Alternative community-modeling methods assume that species evolve to optimize total community growth [38] or to simultaneously optimize their own growth and the community growth [39]. Such assumptions may not be evolutionarily reasonable in various settings but will likely result in markedly more prevalent cooperative behavior. Moreover, our modeling framework can only account for the function of metabolic genes, whereas in reality, a large variety of non-metabolic regulatory mechanisms could potentially impact the evolution of cross-feeding. Another drawback stems from the fact that FBA does not take into account factors such as entropy or pH. For example, the emergence of formate cross-feeding that occurred in our simulations might be less biologically feasible because excess formate accumulation inhibits *E. coli* growth and acidifies the local environment [40]. Another key simplification underlying our work is the assumption of exactly two species that co-exist over a long time scale. This simplification is based on the small population size and drift-dominated evolution in insect endosymbiont systems. More importantly, however, this simplification facilitates many of the analyses reported above, as it enables a clear, rigorous, and well-defined quantification of the impact of one community member on the other. With only two genotypes, the causality and direction of symbiotic relationships is clear, evolutionary emergence of cross-feeding can be compared across trajectories in a fixed context, and the simulation of many replicates is computationally feasible. Finally, following the study reported in [26], our simulations all used *E. coli* as the evolutionary starting point and accordingly some gene- or metabolite-specific results identified in our study may be restricted to this species. We believe, however, that many key patterns observed in the study during the emergence of species interaction would generalize to other bacterial systems.

Future work can further extend our framework or explore the impact of various environmental and evolutionary settings on the emergence of species interactions. For example, most of our analyses above are based on simulating genome reduction as occurring one gene at a time [41], but do not account for the possibility of simultaneous loss of larger genomic regions [42]. Such a process could give rise to different patterns, and indeed our limited analysis of the impact of deleting pairs of genes in each iteration (Additional file 1: Supporting Text) has demonstrated an increase in the prevalence of collapsed communities. Notably, however, a study of a single reductively evolving species that examined both evolutionary regimes did not observe qualitative differences [26]. Similarly, we have demonstrated that using richer media (as opposed to the minimal media used in our primary simulation set to promote cross-feeding) or a different fitness cutoff could have a marked impact on the prevalence of different interactions (see Additional file 1: Support Text). Future work can further explore the effects of different media compositions or of different limiting concentrations on the emergence of species interaction and on the evolving underlying mechanisms in a more systematic and comprehensive manner [43]. A potential future extension of this study could also aim to identify minimal sets of gene deletions that could still give rise to the obligate cross-feeding phenotypes observed in our study after extreme gene loss. Such reduced sets could be, in principle, explored experimentally to validate specific cross-feeding behaviors or more general trends observed in our study. It would also be interesting to expand our framework and to model the evolution of more complex communities (which we did not do here in consideration of simulation time) or to account for spatial heterogeneity [44, 45].

## Conclusion

Using our model, we identified frequent emergence of metabolic dependencies (under specific environmental and evolutionary settings) despite selfish evolution of each species, and we further identified genes and metabolites involved in such evolved cross-feeding interactions. Looking forward, the framework presented in this study could be broadly relevant for improving our understanding of how mutualistic relationships can naturally emerge between bacterial species. This, in turn, would facilitate a deeper understanding of both simple communities, as in the case of insect endosymbionts, and significantly more complex communities, such as those inhabiting the human gut. Moreover, translationally, our approach could be useful to aid and inform the design of dependencies between bacterial species in order to increase the stability and reliability of synthetically constructed bacterial communities or interventions.

## Methods

### Evolution and growth simulations

Every evolution simulation was initiated with two identical copies of the iAF1260 *E. coli* model [46]. During each step in the evolutionary process, a gene (and all the metabolic reactions that depend on this gene) from one of the two species was selected randomly for deletion. If the fitness effect of this deletion in the context of the community (using the co-culture growth model described below) was smaller than the chosen cutoff (5%, as in [26]), the deletion became permanent and the process repeated with the reduced model. Otherwise, the deletion was considered too harmful to occur and the process repeated until a gene that could be deleted was found. The evolutionary process continued until no additional genes could be deleted. The co-culture growth simulation was based on a previously introduced dynamic flux balance analysis framework and is described in detail in Ref [28]. Briefly, given a multi-species community inhabiting a shared medium, the framework assumed that at each time step, each species grew optimally given the current concentration of metabolites in the medium, and then updated the abundance of each species and the concentration of metabolites in the medium based on the predicted growth and activity of each species. For the purpose of this study, both species started at a biomass of 0.01 g dry mass in 1 L volume for mono-culture or 2 L for co-culture, resulting in the same cell density for both (which is equal to about 4x10^7^cells per liter for *E. coli*). The species were grown on a medium based on M9 minimal media [47]. A low concentration (0.0001 mM) of ‘jumpstart’ metabolites were also included to allow growth of obligate mutualistic pairs. Each co-culture simulation consisted of 8 steps of 0.125 h followed by 4 steps of 0.5 h. Additional co-culture growth simulations were performed on the resulting minimal models using a finer time resolution and until the medium was exhausted to confirm that evolved interactions were stable and consistent. For a more detailed description of the evolutionary simulation, the co-culture growth model, and the media see Additional file 1: Supporting Methods.

### Determining interaction types and metabolic dependencies

Interaction type was determined by comparing the fitness of each species when grown in co-culture with its fitness when grown in mono-culture, to assess whether the species was dependent or independent. Communities were labeled as independent, commensal, or mutualistic based on the relationships between the two species. The metabolites (if any) a species depends on were determined by first identifying metabolites that were exchanged between the two species at the final co-culture growth time, and then assaying the growth of each dependent species on minimal medium supplemented with all possible combinations of these exchanged metabolites. If no combination of supplement metabolites allowed such growth the search was expanded to include all combinations of metabolites present in the medium at the end of the co-culture simulation and that were not part of the minimal media (to account for metabolites excreted by the provider at previous time steps). Simulations with ambiguous metabolite dependencies were excluded from metabolite analyses. Additional details can be found in the Additional file 1: Supporting Methods.

### Analyzing evolved genomes and gene retention/deletion

The Jaccard similarity coefficient was used to measure the similarity of two genomes (e.g., in an evolved community). A hypergeometric test was used for determining whether a pair of genes has been co-retained significantly more or less often than expected by chance (at 1% FDR). This analysis was limited to gene pairs that were both retained and deleted at least three times and genes were grouped into sets of perfectly co-varying genes for efficient calculation. Test for enrichment of shared pathways among significant gene pairs was done by permuting the links between pairs. To identify significantly common ordered pairs of gene deletions (at 1% FDR), the number of times gene A in the dependent was deleted before gene B in the provider was recorded and compared to the number observed once the times of deletion (i.e., the positions in the ordering of all gene deletions in that simulation) were permuted.

### Identifying gene-metabolite associations

To identify associations between retention or deletion of specific genes and metabolic phenotypes, the frequency of deletion of a given gene in commensal species that are dependent on a given metabolite was compared to the frequency of deletion of that gene in independent species. This was repeated for commensal species that provided the metabolite their partner depends upon. In both cases, genes being deleted more often or retained more often in species with that metabolic phenotype were identified (at 1% FDR). Only genes that were both deleted and retained at least 10 times and metabolites that were depended upon at least 10 times were considered. These cutoffs were chosen to restrict analysis to genes and metabolites for which sample size would provide statistical power to confidently identify significant correlations.

## Additional files

Additional file 1: Supporting Text, Supporting Methods, **Figures S1-S4.** (683 KB)

Additional file 2: **Table S1.** (115 KB)
