# Supplementary material for: Metabolic model-based analysis of the emergence of bacterial cross-feeding via extensive gene loss
Source: BMC Syst Biol. 2018 Jun 15;12:69. doi: 10.1186/s12918-018-0588-4 (PMC6003207; doi:10.1186/s12918-018-0588-4)
Supplement: Supplementary file 1 — Supporting Text, Supporting Methods, Figures S1-S4. (683 KB) [file 12918_2018_588_MOESM1_ESM.pdf]

Metabolic Model-Based Analysis of the Emergence of Bacterial Cross-Feeding via Extensive Gene Loss

Supporting Materials

Supporting Text

Effect of Fitness Cutoff on Cross-Feeding Prevalence

One parameter that may have a major effect on the emergence of species interaction is the fitness drop cutoff used when allowing a mutation to fix. Presumably, using a less stringent cutoff (i.e., allowing even more deleterious mutations to fix) may allow species to explore additional regions of the fitness landscape and potentially other configurations through which the two species may rely on each other. Conversely, a more stringent cutoff (allowing only very slightly deleterious mutation to fix) may prevent the evolutionary process from reaching configurations that allow the two species to interact. To test this hypothesis, we ran additional simulations, using a cutoff of either 10% or 1% (compared to the 5% used in the main text). Indeed, we found that using a more relaxed cutoff (10%) resulted in a higher frequency of mutualistic, commensal, and collapsed communities, and less independent communities (6.3%, 46.4%, 13.4%, 33.9%, respectively, of a total of 1000 simulation runs; $P < 0.007$ for collapse and $P < 10^{-8}$ for the rest; χ^2 test). Similarly, simulations with a more strict cutoff (1%) had significantly fewer evolutionary trajectories resulting in mutualistic, commensal, or collapsed communities, and more trajectories resulting in independent communities (0%, 27.5%, 3.1%, 69.4%, respectively, of a total of 2000 simulation runs; $P < 10^{-10}$ in all cases; χ^2 test).

Notably, the fitness cutoff used in our study was chosen as an intermediate value between the two cutoffs used by [1], and as shown above has a significant effect on the frequency of evolved commensalism and mutualism. Since this fitness cutoff represents the strength of selection, a permissive

fitness cutoff (as the one used in our study) allows genetic drift to play a dominant role in determining evolutionary trajectories, in agreement with the balance between selection and genetic drift hypothesize to govern the evolution of endosymbionts [2].

Effect of Richer Media on Cross-Feeding Prevalence

Another factor that may likely affect the prevalence at which obligate cross-feeding emerges is the media on which the community grows. Media that is richer than the minimal media used in our primary simulations may inhibit the establishment of cross-feeding since the evolving species will be able to get required resources from the environment without interacting with their partner. Accordingly, we expect that evolution on richer media would increase the prevalence of independent communities at the expense of commensal, mutualistic, and collapsed communities.

To investigate this potential impact of richer media, we first ran 500 additional simulations using a carbon rich media (note that the number of simulations used in this section is substantially smaller than that used in the primary text due to time constraints). This media was similar to the minimal media used in our primary set of simulations but contained five different monosaccharides: glucose, fructose, galactose, mannose, and ribose (in contrast to the minimal media that contain glucose as the sole carbon source). We found that simulations using this carbon rich media in fact resulted in a similar prevalence of independent and mutualistic communities as that observed in simulation on minimal media (52.6% vs. 51% and 2.2% vs. 3%, respectively; $P > 0.05$; χ^2 test). This media did impact however the prevalence of collapsed and commensal communities, resulting in a significant depletion of collapsed communities compared to simulations using the minimal media (5% vs. 10.7% on minimal media; $P < 10^{-4}$) and more commensal communities (40.2% vs. 35.3%; $P < 0.04$; χ^2 test). This finding suggests that the availability of diverse carbon sources may allow communities that would otherwise collapse to survive owning to these additional nutrients in the environment but does not on its own give rise to a significant increase in independent communities.

To explore the effect of other rich media, we further ran simulations using three types of amino-acid rich media. The first contained (in addition to the content of the minimal media) the nine amino-acids that are essential in humans (histidine, isoleucine, leucine, lysine, methionine, phenylalanine, threonine, tryptophan, and valine). The second similarly contained in addition to the minimal media the eleven conditionally or non-essential amino-acids in humans (alanine, arginine, aspartate, asparagine, cysteine, glutamate, glutamine, glycine, proline, serine, and tyrosine). The third contained all twenty amino-acids. We first ran 300 simulations using either the essential amino-acids or the non-essential amino-acids media. We found that these media indeed resulted in a decrease in obligate cross-feeding and an increase in independent communities relative to minimal media. Specifically, simulations using the essential amino-acids media resulted in more independent communities (64.7% vs. 51%; $P < 10^{-5}$) and fewer collapsed communities (1.7% vs. 10.7%; $P < 10^{-6}$). The prevalence of commensal and mutual communities was generally similar to that observed in minimal media (31.7% vs. 35.3 and 2% vs. 3%, respectively). Simulations using the non-essential amino-acids media similarly resulted in more independent communities than minimal media (66.7% vs. 51%; $P < 10^{-7}$), fewer commensal communities (26.7% vs. 35.3%; $P < 0.01$), fewer mutualistic communities (0.3% vs. 3%; $P < 0.01$) and fewer collapsed communities (6.3% vs. 10.7%; $P < 0.05$). Finally, we ran 300 simulations using the media that contained all amino-acids. As expected, this very rich media resulted in dramatically different prevalence of the various interaction types, with markedly more independent communities compared to simulations on minimal media (94% vs. 51%, $P < 10^{-10}$), significantly less commensal communities (6% vs. 35.3%; $P < 10^{-10}$), and no mutualistic or collapsed communities (0% vs. 3%; $P < 0.01$ and 0% vs. 10.7%; $P < 10^{-8}$, respectively). This striking result suggests that the presence of all amino-acids in the media was sufficient to drastically reduce the frequency of dependence evolving.

Effect of Genomic Evolution Pattern on Cross-Feeding Prevalence

In addition to the media, it is also possible that the pattern by which gene deletions occur could affect the likelihood with which obligate cross-feeding evolves in our simulations. Specifically, we wished to

examine whether a non-symmetric deletion rate (i.e., when one species is more likely to have genes deleted than the other) or a multi-gene deletion strategy (i.e., having more than one gene deleted at a time) would impact our findings. To explore this, we ran additional simulation sets (again, using a more limited number of simulation rans) that employ different strategies for choosing which genes to delete. In the first set, we added a bias to the process of selecting which gene to delete, whereby genes in one species were twice as likely to be deleted as the genes in the other species. Running 300 simulations with this strategy we found that the prevalence of the various interaction types was similar to that observed in our main simulation set (independent: 51%, commensal: 34.7%, mutualistic: 2.7%, collapsed: 11.7%; $P > 0.5$ for all compared to unbiased deletions). We additionally did not find significant differences in the average genome size of the deletion-prone species compared to the other ($P > 0.5$, two sample t-test). In the second simulation set, we modeled deletion of larger portions of the genome by deleting two adjacent genes at a time (i.e., pairs of genes that are next to each other based on genomic position). Once no two-gene deletions are possible (i.e., due to large fitness effects) the simulation switches back to deleting genes one at a time (as in our primary simulation) until the minimal genomes are reached. Running 300 simulations using this alternative deletion method, we found that it resulted in fewer independent communities (42.7% vs. 51%; $P < 0.01$) and more collapsed communities (16.7% vs. 10.7%; $P < 0.001$). The prevalence of commensal and mutualistic communities was similar to that observed in our primary simulation set (39.3% and 1.3%, respectively). This finding suggests that deletion of larger portions of the genome at a time could increase the frequency of cross-feeding dependence but could also destabilize such relationships.

Genome Size of Species Evolved in Mono-Culture Conditions

To study the effect of the co-culture condition on the size of the evolved minimal genomes we performed additional simulations in mono-culture growth conditions. Specifically, we ran 2000 evolution simulations that were similar to the simulations described in the main text except that they included only a

single species. We found that the resulting minimal genomes of these species were similar in size to that of species in independent pairs (297.87 vs. 297.85), with no statistically significant difference in size ($P > 0.5$, two sample t-test). As with the independent species' genomes, the mono-culture species' genomes were significantly smaller than commensal provider genomes ($P < 10^{-6}$) and significantly larger than commensal dependent genomes ($P < 10^{-20}$).

Associating Gene Retention with Interaction Type:

We compared mutualistic and independent species and identified a set of 80 genes that are deleted at a significantly higher frequency in mutualistic species (chi-square test; FDR 1%; Table S1B). This set was enriched for various pathways, including oxidative phosphorylation, histidine metabolism, lipopolysaccharide biosynthesis, and nitrogen metabolism. For example, the gene *tyrA*, necessary for tyrosine synthesis, was never deleted in independent species but in 31.9% of mutualistic species, reflecting frequent tyrosine cross-feeding in mutualistic communities (as also observed above). We additionally identified a set of 132 genes that are deleted at a significantly higher frequency in independent species, though this set was not significantly enriched for any specific pathway. Interestingly, however, the gene with the greatest difference in retention rate in this set was *tyrP* (deleted in 96.3% of independent species but only in 68.2% of mutualist species) which is necessary for tyrosine transport, reflecting the need of mutualistic species to uptake this metabolite. Moreover, the larger number of genes identified to be deleted more often in independent species, despite the fact that independent species were found to retain a larger number of total genes compared to mutualistic species, suggests that a multitude of mutualist strategies exist, each involving the retention of a different subset of genes.

Similarity of Retained Gene Sets between Community Partners

We examined how similar, on average, are the sets of genes retained between the two partners in each community. We found that mutualistic species were less similar to each other than independent species ($P < 10^{-20}$; two sample t-test). This finding suggests that the evolution of metabolic dependency is associated

with a process of functional diversification, where each of the two species retains certain metabolic capacities that the other species has lost. This result is perhaps not surprising given the phenotypic differences that must emerge to give rise to mutual dependence on cross-feeding. To further investigate this diversification process we turned our attention again to commensal communities, in which the two species can be labeled as dependent and provider and therefore the direction of dependency is clear. Such communities also represent an intermediate level of interaction as compared to mutualistic communities, in which each species is acting as both provider and dependent, which complicates dissection of the mechanism of interaction. Indeed, the two partner species in commensal communities were more similar to one another than species in mutualistic communities ($P = 1.4 \times 10^{-7}$) but more divergent than species in independent communities ($P < 10^{-20}$).

The Dynamics of Gene Deletion Events

We set out to examine the dynamics of gene deletion events in commensal communities, specifically focusing on the *order* in which deletions occurred in the provider and dependent species and aiming to detect dependencies between these deletion events that could highlight key evolutionary steps on the route to cross-feeding. To this end, we used a permutation-based analysis to identify instances where a gene in one species tended to be deleted after another gene was deleted in the partner species (see Supporting Methods). We identified 9 such gene pairs (at 1% FDR), all of which involved one gene in the provider tending to be deleted first before a second, different gene was deleted in the dependent. Specifically, deletion of the *tyrA* gene in the dependent often followed deletion of a set of genes (*talA*, *talB*, *aroP*, and *pheP*) in the provider. *talA* and *talB* catalyze a reaction connecting glycolysis to the pentose phosphate pathway, and their deletion likely disrupts central carbon metabolism and diverts excess flux toward aromatic amino acid biosynthesis. Similarly, *aroP* and *pheP* are both transporters capable of transporting phenylalanine, and their deletion potentially prompts the excretion of tyrosine instead of phenylalanine. These deletions therefore promote over production and excretion of tyrosine by the provider, allowing the

dependent to lose *tyrA*, a gene necessary for tyrosine synthesis. The deletion of the *pheA*, a gene necessary for phenylalanine synthesis in the dependent, was also found to follow the deletion of *talA* and *talB* in the provider, which is not surprising given the similarity in the biosynthesis pathways of these two amino acids. Finally the deletion of *pyrG* in the dependent tended to follow the deletion of *cdd*, *cmk*, and *codA* in the provider. The deletion of *cmk* (necessary for recycling CMP into CTP) and of *cdd* and *codA* (catalyzing reactions that could convert CMP or related products into other bases) could result in cytidine excretion and accordingly allows the dependent to lose *pyrG* (a component of CTP synthase) which creates a dependency on cytidine (Fig S2). To further examine the mechanism involved in these interactions, we tested whether the deletions of these key genes are sufficient to cause over-production and excretion of the relevant metabolites. Indeed, we found that deletion of *cdd*, *cmk*, and *codA* in the ancestral species (i.e., without any additional gene deletions) led to cytidine excretion. Deletion of *talA, talB, aroP, and pheP* in the ancestral species, however, was insufficient to cause excretion of either phenylalanine or tyrosine, suggesting that additional gene deletions are necessary to give rise to this phenotype.

The Number of Metabolites Excreted by Species Throughout Evolution and its Association with Evolved Interactions

We examined the total number of different metabolites being excreted by each species over time, hypothesizing that species that excrete useful metabolites early on are more likely to become provider species. Surprisingly, however, we found that during the first half of the evolutionary process future providers in fact tend to excrete a similar or even a smaller number of metabolites on average compared to future dependents (Fig S3), and only toward the end of the evolutionary process did providers excrete more metabolites than dependent species. This pattern could suggest that species that eventually became dependent were less optimal early on, excreting more waste products, and that this wasteful behavior may have led to the development of dependence. Notably, all species gradually excrete more metabolites over

the course of the evolution process, likely reflecting more complex growth strategies imposed by their shrinking genome.

Supporting Methods

Evolution Simulation

The evolution simulation was initiated with a pair of genome scale metabolic models. For this study all simulations were initiated with two identical copies of the iAF1260 *E. coli* model [3]. This model includes 1260 genes, 2382 reactions, and 1668 metabolites (which includes extracellular, periplasmic, and cytoplasmic versions of some of the same metabolites). For 304 of those metabolites the model contains transport and exchange reactions that allow them to be exchanged with the external environment. During each step in the evolutionary process, a gene in one of the two species was chosen uniformly at random from the set of all genes still retained by the two species. The chosen gene and all the metabolic reactions that can no longer be performed without this gene were deleted from the species' model. The iAF1260 model contains genes that are required for multiple metabolic reactions as well as genes with redundant effects, and as a result the set of reactions that will be lost as a result of a gene deletion is contingent on which genes have already been deleted previously. The fitness effect of this gene deletion in the context of the community was determined using the co-culture growth model (see below) to evaluate the growth rate of the reduced model when grown with the current model of the partner species. If the calculated fitness effect (when compared to the fitness of that species prior to this gene deletion) was positive, neutral, or smaller than the chosen cutoff (cutoffs used include 1%, 5%, and 10%), the deletion became permanent and the process repeated with the reduced model. However, if the fitness effect exceeded the cutoff, the deletion was considered to be too harmful to occur and the process repeated until a gene that could be deleted was found. This evolutionary process continued until deletion of any remaining gene from either of the two species would cause a drop in fitness exceeding the cutoff, in which case the simulation ended. The simulation also ended if the chosen gene deletion in one species (i.e., a gene deletion that was relatively harmless for that species) caused the other species to drop significantly in

fitness (>50%) in the co-culture. Such simulations, where a partner species was no longer being supported, represent collapsed communities.

Co-Culture Growth Simulation

The co-culture growth simulation was based on a previously introduced dynamic Flux-Balance Analysis framework and is described in more detail elsewhere [4]. Briefly, given a multi-species community inhabiting a shared medium, the framework assumed that at each time step, each species grew optimally given the current concentration of metabolites in the medium (i.e., selfish growth), and then updated the abundance of each species and the concentration of metabolites in the medium based on the predicted growth and activity of each species. Specifically, at each time step, the framework first calculated the upper bound on metabolites' uptake for each species based on the concentration of metabolites in the medium and the cell density of each species. A Flux Balance Analysis (FBA) was then used to determine the fluxes through each species' reactions given these uptake constraints by maximizing the species' biomass production (as a proxy for growth). A second optimization was performed to minimize the total flux through all reactions while keeping the biomass production fixed at the maximum rate (representing a minimization of enzyme usage). The predicted growth rate of each species and the predicted rates at which each species uptakes and excretes various metabolites were then used to update the cell density and concentrations of metabolites in the medium. The process was then repeated at the next time step.

For the purpose of this study, each co-culture simulation consisted of 8 steps of 0.125 hours followed by 4 steps of 0.5 hours. This provided a more accurate account of species growth at the initiation of any potential interaction, while still providing information about the co-culture growth at a longer time scale. The growth rates at the last time point (i.e., after 2.5 hours) were used as a measure of each species' fitness. Both species started at a biomass of 0.01 grams dry mass in 1L volume for mono-culture or 2L for co-culture, resulting in the same cell density for both (which is equal to about 4*10^7 cells per liter for *E. coli*). The species were grown on a medium based on M9 minimal media [5], containing sodium, chloride,

sulfate, inorganic phosphate, potassium, magnesium, ammonia, glucose, water, hydrogen, and oxygen. In addition the metals copper, iron, molybdate, manganese, zinc, nickel, and cobalt were included as they are necessary for growth of the *E. coli* model. These metabolites were all present in the medium at an excess concentration of 10M to ensure exponential growth for the entire course of the co-culture simulation. A low concentration (0.0001 mM) of jumpstart metabolites were also included to allow growth of obligate mutualistic pairs (see below). FBA solutions were calculated using glpkmex, a Matlab interface for GLPK, GNU Linear Programing Kit. GLPK version 4.54 was used, and glpkmex version 2.11.

Jumpstarting Mutualistic Growth

Simulating the growth of species that evolved to be obligate mutualists with a dynamic FBA model has the inherent problem that neither species is able to grow initially on the minimal medium (and consequently will not excrete any of the byproducts needed to allow the other species to grow). In biological systems this problem can be overcome by heterogeneity in the growth phenotypes of individual cells, nutrients released by dead cells, or trace nutrients present in the environment. Rather than simulating diverse growth phenotypes or cell death, in this study we jumpstarted mutualistic growth by supplementing the minimal growth medium described above with trace amounts of potentially necessary metabolites. The set of these "jumpstart metabolites" was determined by identifying metabolites that could be produced by non-transfer reactions still present in at least one of the two species (even if the pathway was not complete). This set therefore represented an upper bound on which metabolites could be exchanged. Jumpstart metabolites were initialized at a low concentration of 0.0001 mM. To ensure that species that utilized these metabolites for growth could eventually be supported by the production of these metabolites by the partner species (rather than continually relying on the trace amounts of these metabolite provided at the beginning of the simulation), at 1 hour into the growth simulation, this same low concentration (0.0001 mM) was subtracted from each jumpstart metabolite.

Filtering Completed Simulation Runs

Simulation runtime considerations necessitated using a relatively limited time resolution in the co-culture growth simulation (see above). To confirm that the growth patterns observed in evolved communities (including obligate cross-feeding interactions) were not artifacts caused by this limited time resolution, for each completed simulation we ran additional co-culture growth simulations on the resulting minimal models using a finer time resolution. Specifically, co-culture growth was simulated until the medium was exhausted with time steps of 0.1 hours, using otherwise the same conditions as the co-culture growth model employed during evolution (including removing the jumpstart metabolites at 1 hour). Community growth was deemed to have been accurately simulated if:

1. Glucose eventually ran out, indicating that the two species were able to continue growing stably.
2. Both species were able to continue growth until this exhaustion of the media. Growth of both species must have been at least 50% of their measured fitness value within the last hour before all growth ended.
3. The growth rate of both species at 2.5 ± 0.2 hours was at least 90% of their fitness value as measured during the course of the evolution.

Simulations that failed any of these three criteria were excluded from the downstream analysis. Of the 16377 completed simulation runs, 16, 317 (99.6%) passed this filtering step. Examples of an evolved mutualistic community that passed this filtering step and an evolved commensal community that failed this filtering step are illustrated in Figure S4.

Simulation Runtime

Each simulation (i.e., a single evolutionary trajectory) required on average 12.2 (± 3.7 STD) hours on a single CPU core. This rather extensive runtime is the outcome of the many FBA estimations performed in each such simulation. Specifically, it should be noted that not only did each ‘generation’ (i.e., gene deletion) in the evolutionary trajectory require 24 FBA optimizations (12 time points for each species) to

obtained the necessary data, but that in fact for each generation our framework may have first tested several potential gene deletions that proved too deleterious and therefore were not selected (see Methods). This step was extremely time-consuming, particularly toward the end of the evolutionary trajectory when more and more (and eventually all) remaining genes *cannot* be selected for deletion. As a result, a single non-collapsed evolutionary simulation required on average ~6,650 co-culture simulations (each involving 24 FBA optimizations), as well as ~1925 additional mono-culture simulations for each of the two species (each involving 12 FBA optimizations for each), resulting in ~205,800 FBA optimization per evolutionary simulation. Simulations were run on a shared high performance computing cluster containing 23 Intel E5345 or E5410 CPUs with 8 cores each, 3 AMD Opteron 6168 CPUs with 48 cores each, and 3 AMD Opteron 6278 CPUs with 64 cores each. Around 80-100 simulations were run simultaneously through the cluster's job scheduling system, and 3 GB of memory was requested for each simulation. The complete set of simulations took about 3-4 months to run.

Determining Interaction Type

Interaction type was determined by comparing the fitness of each species when grown in co-culture with its fitness when grown in mono-culture. If the fitness of a species at a given time point was zero in mono-culture and non-zero in co-culture, the species was labeled as dependent at that time. If it had non-zero growth in both mono- and co-culture it was labeled as independent. Communities were labeled by the relationships between the two species: If both species were independent, the community was labeled as independent. If one species was dependent and the other was independent, the community was labeled as commensal. If both species in a community were dependent, the community was labeled as mutualistic. Within commensal communities, the dependent species was referred to as 'dependent' and the independent species was referred to as 'provider'.

Determining Metabolic Dependencies

For each evolved species we determined what metabolites it depends on (if any). To this end, we first identified metabolites that were being exchanged between the two species at the final co-culture growth time point by finding exchange reactions for which the two species had fluxes of opposite sign. The growth of each dependent species in the pair was then assayed on minimal medium supplemented with all possible combinations of these exchanged metabolites, using a single time step mono-culture growth model, to identify the smallest set of supplement metabolites that allowed it to grow at >50% its growth rate in co-culture. If no combination of supplement metabolites allowed such growth the search was expanded to include all combinations of metabolites present in the medium at the end of the co-culture simulation and that were not part of the minimal media (such metabolites could have been excreted by the provider at previous time steps). In 359 simulations multiple sets of supplement metabolites were sufficient for growth and were neither subsets nor supersets of each other. These ambiguous simulations were excluded from any analysis of metabolite dependency or association.

Pathway Analyses

Pathways annotations for each gene in the model were obtained from the Kyoto Encyclopedia of Genes and Genomes (KEGG) [6]. To identify enrichment of KEGG pathways in subsets of genes (e.g., those that were deleted at significantly different rates between interaction types), we generated 100,000 random subsets (out of the 551 genes retained at intermediate frequencies) of the same size and compared the total number of genes associated with each pathway in the real set to the number of genes associated with that pathway in random sets.

Measuring Genome Similarity

To compare the similarity of two genomes (e.g., in an evolved community), we used the Jaccard similarity coefficient. We then used a two-sample t-test to test for significant differences in similarity between different types of communities.

Identifying Co-Retained Gene Pairs

To identify gene-gene co-occurrence relationships, we examined all pairwise combinations of genes that were both retained and deleted at least five times. This cutoff was chosen to restrict analysis to genes for which sample size would provide enough statistical power to confidently identify co-occurrence. Since many genes perfectly co-varied with each other across simulations, we first grouped genes into sets of perfectly co-varying genes and identified co-occurrence relationships between these sets. For each pair of gene sets, we found the number of species that had retained each set and the number of species that had retained both sets and used a hypergeometric test to determine whether these sets have been co-retained significantly more or less often than expected by chance (at 1% false discovery rate; [7]). Test for enrichment of shared pathways among the significant gene set pairs was done by permuting the connections between pairs.

Identifying Significant Gene Deletion Ordering

Given a pair of genes, A and B, we recorded the number of times gene A in the dependent was deleted before gene B in the provider. We then used a permutation-based assay, permuting the time of deletion (measured as the position in the ordering of all gene deletions in that simulation) of each gene between all providers or dependents from commensal communities in which that gene had been deleted. Gaps and overlaps in the resulting permuted gene deletion histories were resolved by shifting deletions into gaps and randomly breaking ties. The number of times gene A in the dependent was deleted before gene B in the provider in the original data was compared to this number in the permuted data to identify significantly common ordered pairs of gene deletes (at 1% false discovery rate).

Gene-Metabolite Connections

To identify correlations between retention or deletion of specific genes and metabolic phenotypes, we considered all genes that were both deleted and retained more than 10 times and all metabolites that were

depended upon at least 10 times. These cutoffs were chosen to restrict analysis to genes and metabolites for which sample size would provide statistical power to confidently identify significant correlations. For every pair of such genes and metabolites, we compared the frequency of deletion of that gene in commensal species that are dependent on that metabolite to the frequency of deletion of that gene in independent species. This was repeated for commensal species that provided the metabolite their partner depends upon, and in both cases instances of genes being deleted more often or retained more often in species with that metabolic phenotype were identified (at 1% false discovery rate).

Supporting References

1. Pál C, Papp B, Lercher MJ, Csermely P, Oliver SG, Hurst LD. Chance and necessity in the evolution of minimal metabolic networks. Nature. 2006;440: 667–70. doi:10.1038/nature04568

2. Bennett GM, Moran NA. Heritable symbiosis: The advantages and perils of an evolutionary rabbit hole. Proc Natl Acad Sci. 2015;112: 10169–10176. doi:10.1073/pnas.1421388112

3. Feist AM, Henry CS, Reed JL, Krummenacker M, Joyce AR, Karp PD, et al. A genome-scale metabolic reconstruction for Escherichia coli K-12 MG1655 that accounts for 1260 ORFs and thermodynamic information. Mol Syst Biol. 2007;3: 121.

4. Chiu H-C, Levy R, Borenstein E. Emergent biosynthetic capacity in simple microbial communities. PLoS Comput Biol. 2014;10: e1003695. doi:10. 1371/journal.pcbi.1003695

5. Sambrook J, Fritsch EF, Maniatis T. Molecular Cloning: A Laboratory Manual. Second edi. Cold Spring Harbor, New York: Cold Spring Harbor Laboratory Press; 1989.

6. Kanehisa M, Goto S. KEGG: kyoto encyclopedia of genes and genomes. Nucleic Acids Res. 2000;28: 27–30.

7. Benjamini Y, Hochberg Y. Controlling the false discovery rate: a practical and powerful approach to multiple testing. J R Stat Soc B. 1995;57: 289–300.

Supporting Figures

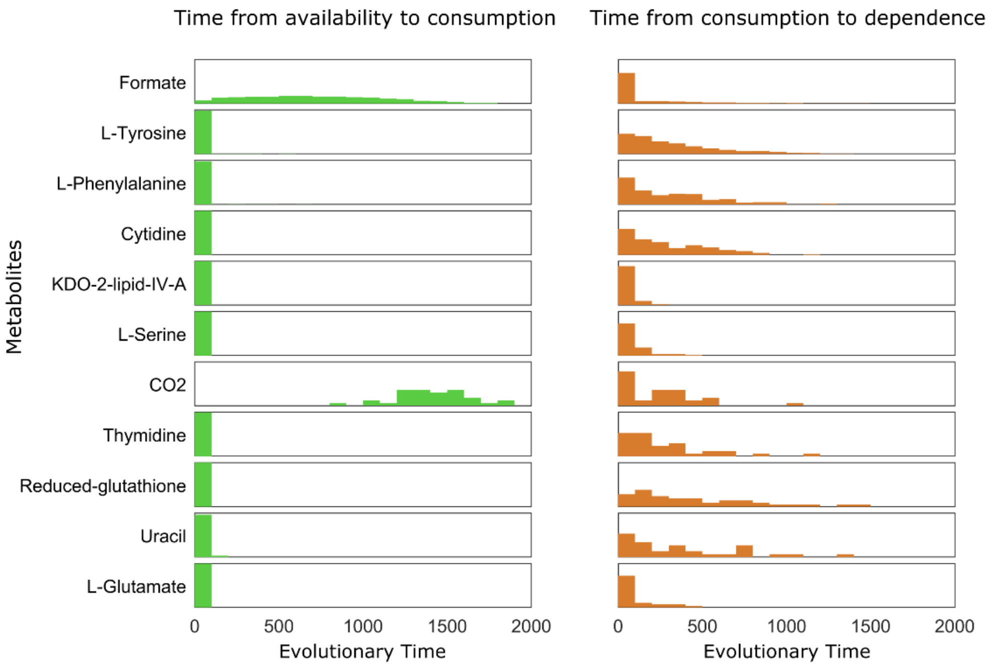

Figure S1: Time of emergence of metabolites' availability, cross-feeding, and dependence. The distributions of evolutionary time (measured as number of gene deletions) elapsed between the different stages of metabolic interaction, for commensal species dependent on a single metabolite.

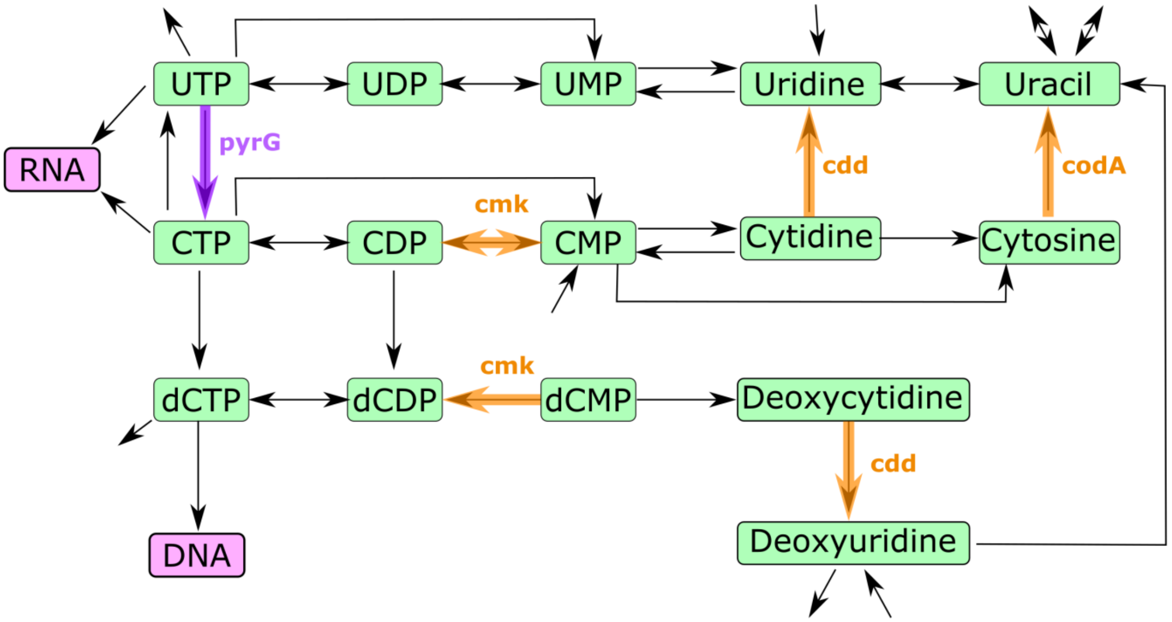

Figure S2: Dependent deletion events in pyrimidine biosynthesis. The subset of the pyrimidine biosynthesis network in the *E. coli* model is shown. Reactions catalyzed by enzymes encoded by genes that tend to be lost first in the provider are marked in orange (*cmk*, *cdd*, and *coda*), and the reaction catalyzed by an enzymes encoded by a gene that is lost later in the dependent is marked in purple (*pyrG*).

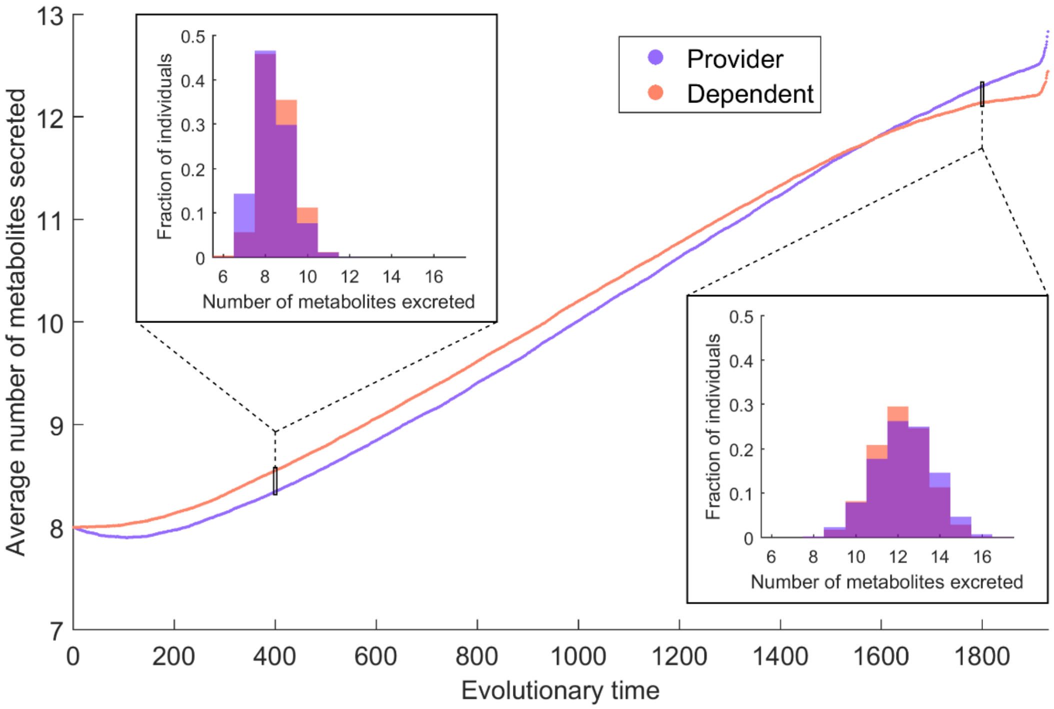

Figure S3: The number of different metabolites excreted by species of different interaction types over evolutionary time. Plotted is the mean number of unique metabolites excreted by dependent and provider species from commensal communities at each point throughout he evolutionary process. The two insets show the distribution of the number of metabolites excreted at time 400 and 1800 for both dependents and providers.

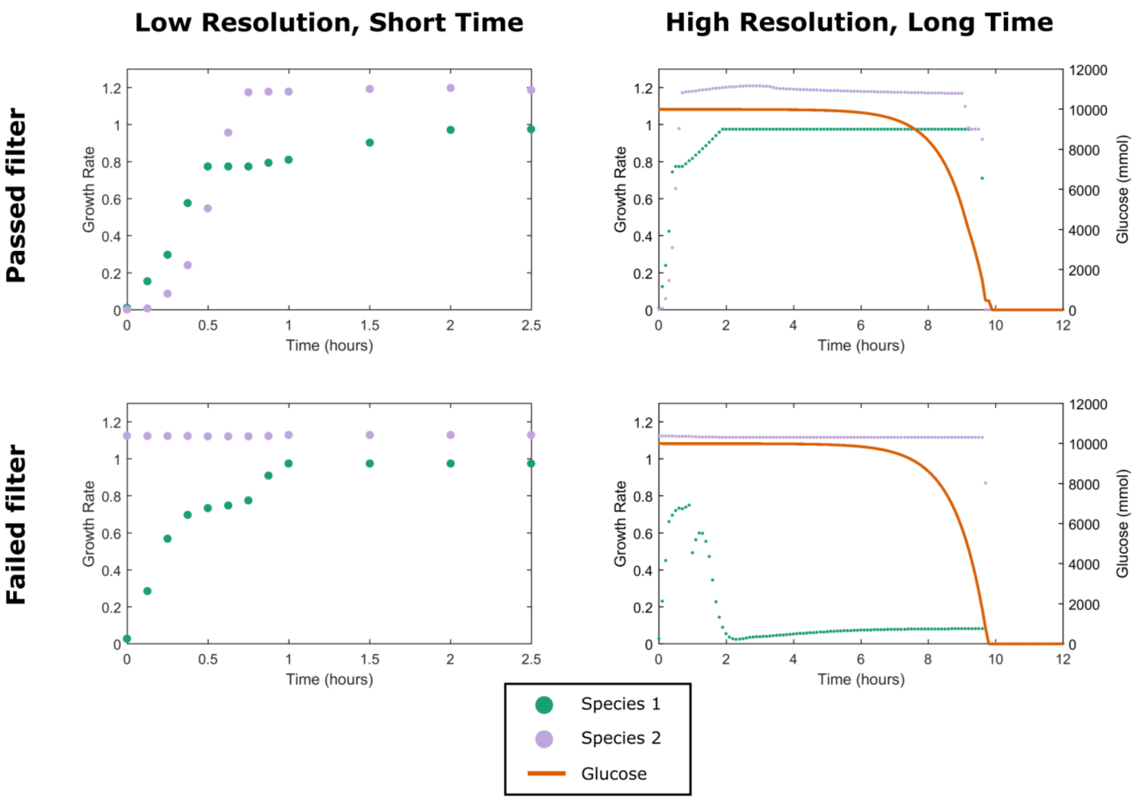

Figure S4: Example of co-culture growth of communities that passed and failed the quality filtering step. The co-culture growth of evolved pairs are plotted both for the settings used during the evolution simulation (left: low resolution, short time) and the settings used to validate growth during filtering (right: high resolution, long time). On the top is an evolved mutualistic community that passed the filter, and on the bottom is an evolved commensal community that failed the filtering step. When the top community was grown with high resolution time steps both species reached similar growth rates to that seen at the last time point of the low resolution simulation, and maintained roughly those growth rates until glucose was exhausted. The bottom community fails to show such consistent behavior. Specifically species 1 never reaches the growth rate it achieved in the low resolution simulation, and instead collapses to a very low growth rate shortly after the jumpstart metabolites are removed (1 hour).
